# *Chaoborus* and *Gasterosteus* Anti-Predator Responses in *Daphnia pulex* Are Mediated by Independent Cholinergic and Gabaergic Neuronal Signals

**DOI:** 10.1371/journal.pone.0036879

**Published:** 2012-05-09

**Authors:** Linda C. Weiss, Sebastian Kruppert, Christian Laforsch, Ralph Tollrian

**Affiliations:** 1 Animal Ecology Evolution and Biodiversity, Ruhr-University Bochum, Bochum, Germany; 2 Department of Biology II, Ludwig-Maximilians-University, Munich, Germany; Federal University of Rio de Janeiro, Brazil

## Abstract

Many prey species evolved inducible defense strategies that protect effectively against predation threats. Especially the crustacean *Daphnia* emerged as a model system for studying the ecology and evolution of inducible defenses. *Daphnia pulex* e.g. shows different phenotypic adaptations against vertebrate and invertebrate predators. In response to the invertebrate phantom midge larvae *Chaoborus* (Diptera) *D. pulex* develops defensive morphological defenses (neckteeth). Cues originating from predatory fish result in life history changes in which resources are allocated from somatic growth to reproduction. While there are hints that responses against *Chaoborus* cues are transmitted involving cholinergic neuronal pathways, nothing is known about the neurophysiology underlying the transmission of fish related cues. We investigated the neurophysiological basis underlying the activation of inducible defenses in *D. pulex* using induction assays with the invertebrate predator *Chaoborus* and the three-spined stickleback *Gasterosteus aculeatus*. Predator-specific cues were combined with neuro-effective substances that stimulated or inhibited the cholinergic and gabaergic nervous system. We show that cholinergic-dependent pathways are involved in the perception and transmission of *Chaoborus* cues, while GABA was not involved. Thus, the cholinergic nervous system independently mediates the development of morphological defenses in response to *Chaoborus* cues. In contrast, only the inhibitory effect of GABA significantly influence fish-induced life history changes, while the application of cholinergic stimulants had no effect in combination with fish related cues. Our results show that cholinergic stimulation mediates signal transmission of *Chaoborus* cues leading to morphological defenses. Fish cues, which are responsible for predator-specific life history adaptations involve gabaergic control. Our study shows that both pathways are independent and thus potentially allow for adjustment of responses to variable predation regimes.

## Introduction

Phenotypic plasticity is the ability of organisms with a given genotype to form different phenotypes in response to changing environmental conditions. E.g. the ability to form inducible defenses under an increased risk of predation increases the chance of survival in a high predation environment but reduces costs when predators are absent [1], [2]. Inducible defenses in the freshwater crustacean genus *Daphnia* have been intensely studied. *Daphnia* form behavioral [3], [4], morphological [5], [6] and life history defenses [6], [7] in response to chemical cues from vertebrate [8] and invertebrate [9] predators. These so-called kairomones are defined as chemical cues in the interspecific information transfer, that are beneficial to the receiver but not to the sender. Cues released by the phantom midge larvae *Chaoborus* (Diptera) e.g. induce neckteeth [5], [9] while cues from fish predominantly induce changes in life history parameters [10], [11]. Whereas the ecology and evolution of inducible defenses have been described in detail in a variety of *Daphnia* species [reviewed in 2], the modes of predator detection and the pathways of signal transmission remained elusive. Moreover, the kairomones released by both predators have not been chemically identified yet. Nevertheless, the distinct differences in defense strategies highly suggest that *Daphnia* must be capable to distinguish between these predator cues.

So far, there is only one study that precisely addressed the neurophysiology of *Chaoborus* kairomone perception [12]. Here the author measured neurotransmitter-dependent neckteeth expression in *D. pulex*. Cholinergic stimulation and gabaergic inhibition was proposed as an essential component of the signaling cascade of kairomone perception.

No information exists on how fish kairomones are perceived and how this information is transformed into life history adaptations. Moreover, the mode of discrimination between both types of predators has not been addressed up to date.

We postulate three hypotheses that might explain the different predator responses. First, fish kairomone perception might not be bound to neurophysiological signals at all. Second, antagonistic inhibition, in which perception of one predator with subsequent defensive phenotype development inhibits the alternative response, seems a probable mechanism. A third option would be an uncoupled system with independent predator-specific physiology.

In order to elucidate the nature of this physiological system we performed an induction assay using different *Gasterosteus* and *Chaoborus* kairomone concentrations in combination with different stimulatory and inhibitory neurotransmitters.

Our results confirm the distinct effects of *Chaoborus* and *Gasterosteus* kairomone exposure on life history and morphological defenses in *D. pulex*. We stimulated and inhibited the gabaergic and the cholinergic nervous system in kairomone presence and absence and measured how these pathways modulate the *Gasterosteus* and *Chaoborus* kairomone specific phenotypic adaptations. We applied the neuro-stimulants GABA and physostigmine. GABA is the natural stimulant of the different GABA receptor types. GABA stimulation in general leads to the hyperpolarization of the cellular membrane and results in inhibitory cellular responses accompanied by decreased neuronal firing rates. Physostigmine inhibits the enzyme acetylcholine esterase in the synaptic cleft. This enzyme degrades acetylcholine bound to the post-synaptic receptors. An inhibition of this enzyme prolongs receptor activation and subsequent excitation of the membrane caused by a prolonged cholinergic binding at either nicotinic or muscarinic receptors with increased neuronal firing rates.

The GABA antagonist picrotoxin inhibits gabaergic transmission at the GABA_A_- receptor. As GABA itself is an inhibitory neurotransmitter, application of picrotoxin, which acts as a non-competitive antagonist, has stimulatory effects.

Atropine is a drug with anticholinergic effects. It acts as a competitive antagonist that inhibits the muscarinic acetylcholine receptor. Thereby cholinergic stimulation is decreased.

The results obtained in our study are a first approach to the understanding of the neurophysiological mechanisms underlying the development of anti-predator responses. We show that *Chaoborus* kairomone perception inducing morphological defenses is bound to cholinergic signals. In contrast, fish kairomone perception inducing life-history shifts are under gabaergic control. Thus, neuronal processing of fish and *Chaoborus* cues acts independently rather than antagonistically.

## Results

### Predator responses

**Figure 1:**
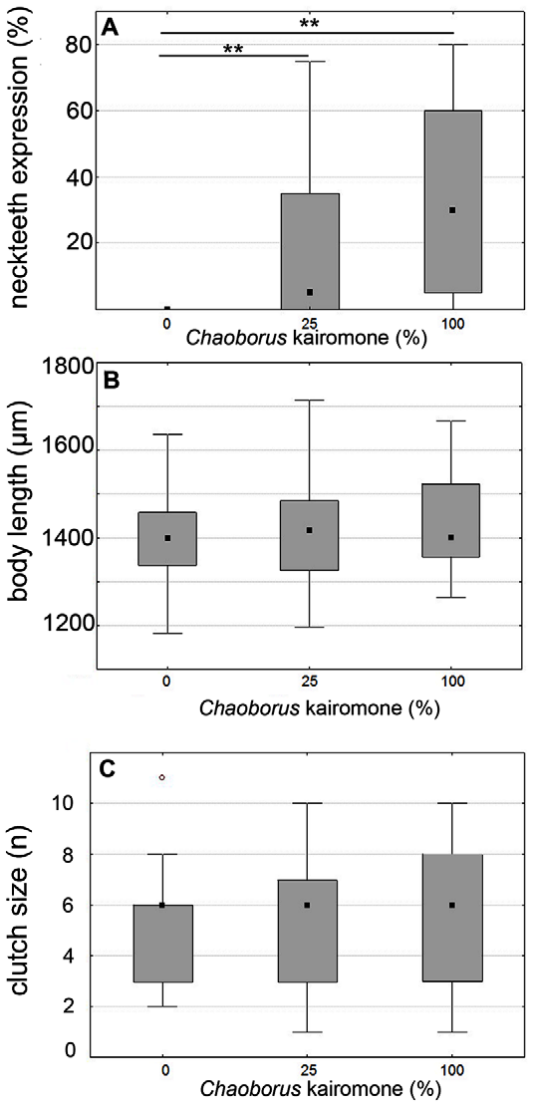
Inducible morphological defenses against *Chaoborus* predation in the second juvenile instar. A: *Chaoborus* kairomone significantly increases neckteeth expression in juvenile *D. pulex*. B and C: No significant impact was observed on life history parameters at sexual maturity of *Chaoborus* exposed *D. pulex.* Body length (B) and number of neonates (C) is not significantly different from control groups. Plotted are medians and interquartile ranges. Bonferroni corrected significance levels: *p<0.025; **p<0.005; ***p<0.0005.

Our clones of D. pulex showed distinct anti-predator responses against chemical cues released by fourth instar *Chaoborus* larvae. We found a significantly enhanced development of neckteeth in *Chaoborus* exposed juvenile *D. pulex* (25% *Chaoborus* kairomone: Mann-Whitney U = 1529; p<0.005; 100% *Chaoborus* kairomone: Mann-Whitney U = 697.5; p<0.005; Fig. 1 A). Life history parameters (body size and number of first clutch neonates) of adult (sexually mature) *Chaoborus* exposed *D. pulex* were not influenced by an increased predation risk (Fig. 1 B, C).

**Figure 2:**
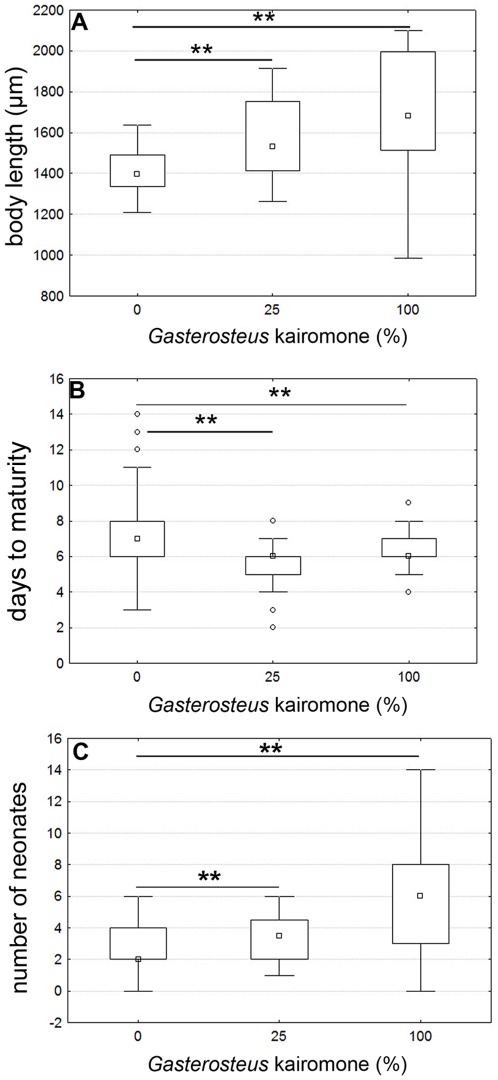
Life history shifts against *Gasterosteus* predatory cues at sexual maturity. A: In dependence of the *Gasterosteus* kairomone the body length is significantly increased. B: Time to reach maturity is significantly shortened and C: The number of neonates is significantly increased. Plotted are medians and interquartile ranges. Bonferroni corrected significance levels: *p<0.025; **p<0.005; ***p<0.0005.

*Gasterosteus* kairomone exposed daphnids did not display neckteeth but showed profound changes in life history parameters (Fig. 2). Body size at sexual maturity was significantly increased already at low kairomone concentrations (25% *Gasterosteus* kairomone: Mann-Whitney U = 416; p<0.005; Fig. 2 A). This result is maintained at high concentrations (100% *Gasterosteus* kairomone: Mann-Whitney U = 475; p<0.005). Furthermore, fish exposed daphnids reproduced faster (25% *Gasterosteus* kairomone: Mann-Whitney U = 381.5; p<0.005; 100% *Gasterosteus* kairomone: Mann-Whitney U = 1128.5; p<0.005; Fig. 2 B) with an increased number of neonates in the first clutch in comparison to un-exposed *D. pulex* (25% *Gasterosteus* kairomone: Mann-Whitney U = 341.5; p<0.005; 100% *Gasterosteus* kairomone: Mann-Whitney U = 589; p<0.005; Fig. 2 C).

### Cholinergic stimulation and inhibition

**Figure 3:**
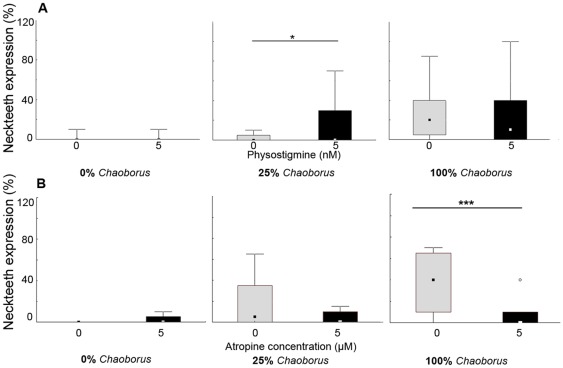
Cholinergically modulated neckteeth expression. Neckteeth expression in dependence of increasing *Chaoborus* kairomone concentrations in combination with (A) physostigmine (5 nM) and (B) atropine (5 µM). Physostigmine significantly increases neckteeth expression at low (25%) kairomone concentrations. At high concentrations a maximal expression of neckteeth cannot be exceeded. Atropine significantly reduces neckteeth expression at high (100%) kairomone concentrations in comparison to lower (25%) concentrations. Plotted are medians and interquartile ranges.

The application of the acetylcholine esterase inhibitor physostigmine showed a significant increase of morphological defenses in response to 25% *Chaoborus* chemical cues (Mann-Whitney U = 1710; p<0.05; Table 1; Fig. 3 A).

**Table 1:** Physostigmine (5 nM) effect on morphological defenses in dependence of *Chaoborus* and *Gasterosteus* kairomone (Bonferroni-corrected Mann-Whitney U comparison).

	2nd juvenile instar									
PHYSOSTIGMINE	*Chaoborus* kairomone					*Gasterosteus* kairomone				
paired groups	0%	25%	50%	100%	Effect	0%	25%	50%	100%	Effect
NT expression	ns	**p<0.05**	**ns**	**p = 0.08**	**in-crease**	ns	ns	ns	ns	none
body length	ns	ns	ns	ns	none	ns	ns	ns	ns	none

	Sexual maturity									
	*Chaoborus* kairomone					*Gasterosteus* kairomone				
paired groups	0%	25%	50%	100%	Effect	0%	25%	50%	100%	Effect
body length	ns	ns	ns	ns	none	ns	ns	ns	ns	none
number of eggs	ns	ns	ns	ns	none	ns	ns	ns	ns	none
days until maturity	ns	ns	ns	ns	none	ns	ns	ns	ns	none

(NT) neckteeth.

(ns) not significant.

(Effect) describes the overall increase or decrease of the observed treatment.

Inhibition of cholinergic stimulation using the muscarinic antagonist atropine resulted in a decreased expression of neckteeth in *Chaoborus* exposed *D. pulex*. Especially at medium (50%; Mann-Whitney U = 422; p<0.05; Table 2) and high (100%; Mann-Whitney U = 147; p<0.001; Table 2; Fig. 3 B) *Chaoborus* kairomone concentrations we noticed an atropine dependent decrease of neckteeth development in comparison to the kairomone controls. Furthermore, *Chaoborus* exposed 2^nd^ juvenile instar *D. pulex* showed an atropine dependent reduction of the body size (50%; Mann-Whitney U = 422; p<0.01 and 100% Mann-Whitney U = 147; p<0.001; Table 2), but this decrease was not consistent throughout ontogenetic development and was not observed at sexual maturity. Additionally, life history parameters including numbers of eggs in the first clutch size and days until maturity measured in sexually mature specimens were not influenced. In combination with chemical cues originating from fish we did not observe any effects of cholinergic stimulation on life history parameters.

**Table 2:** Atropine (5 µM) effect on morphological defenses in dependence of *Chaoborus* and *Gasterosteus* kairomone (Bonferroni- corrected Mann-Whitney U comparison).

	2nd juvenile instar									
ATROPINE	*Chaoborus* kairomone					*Gasterosteus* kairomone				
paired groups	0%	25%	50%	100%	Effect	0%	25%	50%	100%	Effect
NT expression	ns	ns	**p<0.01**	**p<0.001**	**Decrease**	ns	ns	ns	ns	none
body length	**p<0.05**	ns	**p<0.01**	**p<0.01**	**Decrease**	ns	ns	ns	ns	none

	Sexual maturity									
	*Chaoborus* kairomone					*Gasterosteus* kairomone				
paired groups	0%	25%	50%	100%	Effect	0%	25%	50%	100%	Effect
body length	ns	ns	ns	ns	**none**	ns	ns	ns	ns	**none**
number of eggs	ns	ns	ns	ns	**none**	ns	ns	ns	ns	**none**
days until maturity	ns	ns	ns	ns	**none**	ns	ns	ns	ns	**none**

(NT) neckteeth.

(ns) not significant.

(Effect) describes the overall increase or decrease of the observed treatment.

### GABA application

**Figure 4:**
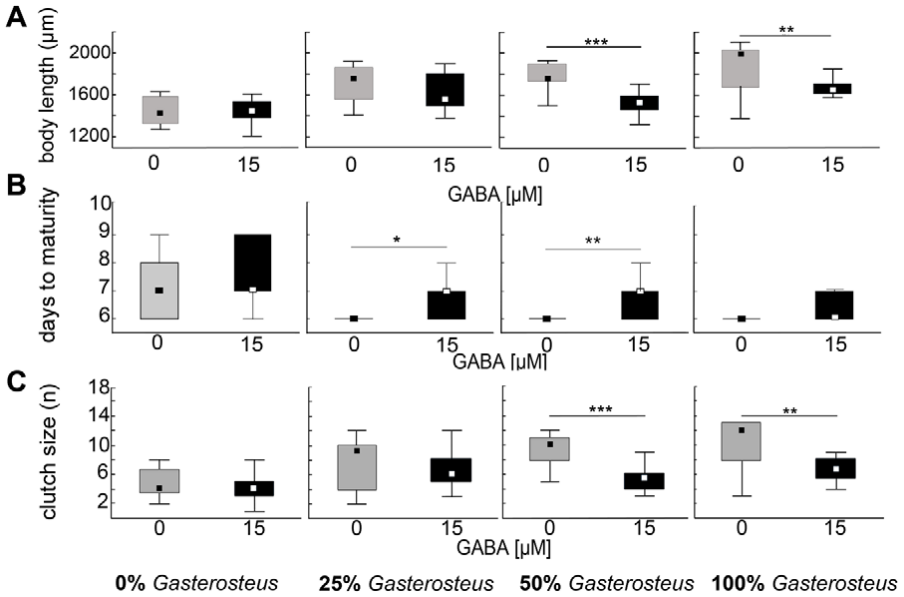
Gabaergically modulated life history parameters. A: Body length in dependence of increasing *Gasterosteus* kairomone concentrations and GABA (15 µM). Body length is significantly reduced by GABA stimulation at high *Gasterosteus* kairomone concentrations (50% and 100%, Mann-Whitney U comparison p<0.01). B: Generation time is significantly increased by GABA stimulation at low and medium *Gasterosteus* kairomone concentrations (25% and 50%, Mann- Whitney U comparison p<0.01). C: The fecundity (number of eggs in the brood pouch) is significantly reduced by GABA stimulation at high *Gasterosteus* kairomone concentrations (75% and 100%, Mann- Whitney U comparison p<0.01).

GABA application was not effective in combination with *Chaoborus* kairomones. In contrast, GABA applied in conjunction with *Gasterosteus* cues showed a significantly decreased body size at low (25%; Mann-Whitney U = 32; p<0.05; Table 3), medium (50%; Mann-Whitney U = 31; p<0.05; Table 3) and high (100%; Mann-Whitney U = 57; p<0.05; Table 3) kairomone concentrations in the 2^nd^ juvenile instar. Life history parameters including body length (50% Mann-Whitney U = 31; p<0.001; and 100%; Mann-Whitney U = 57 p<0.01; Table 3; Fig. 4 A), days until maturity (25%; Mann-Whitney U = 95; p<0.05; 50%; Mann-Whitney U = 51; p<0.001; Table 3; Fig. 4 B) and number of eggs obtained in the first clutch (50%; Mann- Whitney U = 50.5; p<0.001; 100%; Mann-Whitney U = 51; p<0.01; Table 3; Fig. 4 C), of sexually mature specimen were all significantly decreased (Table 3). The GABA_A_ receptor inhibitor picrotoxin did not impact any morphological or life history traits of neither *Chaoborus* kairomone nor *Gasterosteus* kairomone treatments.

**Table 3:** GABA (15 µM) effect on morphological defenses in dependence of *Chaoborus* and *Gasterosteus* kairomone (Bonferroni- corrected Mann-Whitney U comparison).

	2nd juvenile instar									
GABA	*Chaoborus* kairomone					*Gasterosteus* kairomone				
paired groups	0%	25%	50%	100%	Effect	0%	25%	50%	100%	Effect
NT expression	ns	ns	ns	ns	**none**	ns	ns	ns	ns	**none**
body length	ns	ns	ns	ns	**none**	ns	**p<0.05**	**p<0.05**	**p = 0.04**	**Decrease**

	Sexual maturity									
	*Chaoborus* kairomone					*Gasterosteus* kairomone				
paired groups	0%	25%	50%	100%	Effect	0%	25%	50%	100%	Effect
body length	ns	ns	ns	ns	**none**	ns	ns	**p<0.001**	**p<0.01**	**Decrease**
number of eggs	ns	ns	ns	ns	**none**	ns	ns	**p<0.001**	**p<0.01**	**Decrease**
days until maturity	ns	ns	ns	ns	**none**	ns	**p<0.05**	**p<0.001**	**ns**	**Increase**

(NT) neckteeth.

(ns) not significant.

(Effect) describes the overall increase or decrease of the observed treatment.

## Discussion

The *D. pulex* clone in our study has the ability to respond to two different predatory threats. The reaction was very distinct and resulted in a morphological defense against *Chaoborus* predation and shifts in life history parameters against fish predation.

Physostigmine, atropine and GABA were only effective in combination with the respective kairomone cue. None of the applied substances alone initiated the expression of a defense. This indicates that the pathway underlying kairomone perception requires signal transmission involving a cascade of multiple steps [13], [14].

We observed a significant positive modulation of neckteeth expression in dependence of the acetylcholine esterase inhibitor physostigmine. Inhibition of cholinergic stimulation using the muscarinic antagonist atropine resulted in a decreased expression of neckteeth in *Chaoborus* exposed *D. pulex*. Whereas physostigmine significantly increased neckteeth expression at low (25%) kairomone concentrations, atropine significantly reduced neckteeth expression at high (100%) kairomone concentrations. The first effect is explainable because a maximal development of neckteeth resulting from a high concentration of *Chaoborus* cues (50% and 100%) cannot be significantly increased, not even by experimental cholinergic stimulation since every morphological response can only be expressed within its physiological limits. Vice versa, maximal effects can be measurably inhibited by the application of the antagonist. Thus atropine is not notably effective at low (25%) kairomone concentrations however it significantly reduces the effect of (100%) kairomone concentrations.

These results clearly demonstrate a cholinergic influence in the transmission of the *Chaoborus* kairomone signal. Cholinergic stimulation or inhibition did not impact any life history parameters of *D. pulex* induced by fish cues and therefore seems to be specifically responsible for the transmission of morphological defenses that are induced by *Chaoborus* cues. We noticed a significant (p<0.05) atropine dependent decrease of the body length in the second juvenile instar in combination with a low *Chaoborus* kairomone (25%) concentration. This effect however did not occur at sexual maturity, a stage in which life-history traits should be visible. We therefore assume that this effect occurred by chance.

The application of the, in general, inhibitory neurotransmitter GABA (Table 3) did not decrease or inhibit morphological defense structures against *Chaoborus* cues. This indicates that GABA does not play an inhibitory role in *Chaoborus* kairomone perception.

However, GABA modulated life history responses against fish predation. Body length and clutch size were reduced and the time until maturity increased. This indicates the presence of a gabaergic neuronal control underlying the neurophysiology of fish kairomone transmission. The observed responses could potentially be explained by a relieved inhibition, which is reestablished by the experimental application of GABA. In general, GABA is known to have inhibitory functions. In the state without vertebrate predators, *Daphnia* life history shifts could be inhibited by gabaergic signals. This results in a ‘general purpose’ life history in the absence of fish. Upon the perception of fish cues this gabaergic inhibition might be relieved and specific life history parameters change. This would elicit the adjustment of life history parameters in a fast and time-efficient manner. Such a GABA dependent predator perception was just recently shown in larval coral reef fish [15].

We can rule out toxic effects of GABA on life history parameters since GABA was only effective in combination with the fish kairomone and showed no effect in control treatments. Furthermore, it did not influence life history parameters in the *Chaoborus* kairomone treatments and all control treatments.

The gabaergic antagonist picrotoxin did not show any effect in this study. We had expected that picrotoxin enhances neckteeth expression as previously shown [12]. Using the same concentrations we did not observe a comparable effect. Furthermore, we did not observe any picrotoxin effect on life history parameters as a response to fish predation. The overall ineffectiveness of this GABA_A_ receptor antagonist might indicate clonal differences potentially resulting from different receptor amino acid sequences. E.g. GABA receptor subtypes with a single amino acid replacement makes the *Drosophila* GABA_A_ receptor picrotoxin insensitive [16]. Furthermore, crustacean GABA_A_ receptors were found insensitive towards picrotoxin [17].

Our results support the findings of Barry [12] that *Chaoborus*-induced morphological defenses are cholinergically transmitted. Our investigation of the later life stages, however, shows that cholinergic stimulation has no effect on life history parameters. This indicates that cholinergic signaling is only involved in the neuronal pathways of *Chaoborus* kairomone perception and the development of morphological defenses. Cholinergic stimulation and inhibition had no impact on fish kairomone-dependent responses. Thus cholinergic pathways are unlikely to be involved in the perception of fish cues accompanied by life history shifts.

However, GABA receptor stimulation inhibits fish kairomone-induced life history shifts. This provides strong evidence that the nervous system is involved in the physiological pathways underlying the development of fish-dependent physiological responses and that life history parameters induced by fish cues are under the neurophysiological control of GABA. Our study suggests that the GABA inhibition is abolished upon predator perception and reinstalled by GABA application. Hence, our results provide a functional explanation for the physiological effects observed.

### Conclusion

We showed that inducible defenses against *Chaoborus* and fish predators are neurophysiologically coded.

We found no experimental support for an antagonistic system, in which anti-predator responses are physiologically coupled. Since we found two different pathways for *Chaoborus* and fish perception, we conclude that predator-induced defenses in *D. pulex* are not bound to a coupled system but rather depend on two independent neuronal systems. This may be advantageous as it allows for mixed responses, which may have the potential to defend the organism from several predation pressures simultaneously. However, future investigations need to address the effect of two predators present simultaneously and determine their effect on inducible defenses.

The neurotransmitters applied in our experiments were able to modulate the kairomone response. However, without the kairomone stimulus, neurotransmitters were not able to elicit the development of inducible defenses. Thus cholinergic and gabaergic stimulations alone do not activate the defense directly. These results suggest a pathway with multiple steps, which requires some kind of additional neuronal transmission. We were here able to give an initial insight into the neuronal mechanisms of cue perception and interpretation underlying the development of inducible defenses in *Daphnia*.

## Materials and Methods

Animals used for kairomone production were collected from the ponds of the Ruhr-University Bochum and no specific permits were required for the sampling in field sites. These sampling sites are not privately owned or protected in any way. Species collected did not involve endangered or protected species. A study approval by a named review board institution or ethics committee including permit numbers or approval ID has not been received nor has been asked for, because it is irrelevant in our case. There is no ethics committee or review board institution dealing with capture, short term rearing and release of fish and invertebrates from the university ponds. Fish and invertebrate larvae have not been used in our experiments. We only used the rearing water.

### Culture

Induction assays were performed with an institute's laboratory clone of *D. pulex*, which readily responds to predator cues with morphological and life history adaptations [9]. This clone shows maximal neckteeth expression in the second and third instar.

All *D. pulex* specimens were raised in charcoal-filtered tap water and were fed ad libitum with the algae *Scenedesmus obliquus* (1.5 CL^−1^). Animals were cultured in a climate chamber at 20°C±1°C with a 12∶12 hours day∶night cycle. The induction assay was performed in a climate chamber (KBF 720L, Binder GmbH, Germany) at 20°C±0,1°C (12∶12, day∶night cycle) in order to ensure stable temperature conditions.

### Chaoborus sampling and kairomone production

*Chaoborus obscuripes* were sampled from the ponds of the Ruhr-University's Botanical Garden for the production of the kairomone. After the experiment the animals were brought back to the pond of origin.

Fourth instar larvae were isolated from the sampling collection and transferred into 1 L glass beakers (WECK ®) filled with charcoal-filtered tap water. For the production of the kairomone 50 larvae were fed with 500 juvenile *D. pulex* and cultured in 1 L water (Evian, Danone Waters, Germany) for 24 h. Kairomone conditioned medium was prepared by filtration (*Artemia* sieve with mesh size of 50 µm) and stored until use at −20°C in 50 ml falcon tubes. This was defined as 500% kairomone concentration because it was five fold diluted to a maximum concentration of 100% in the induction assay.

### Gasterosteus *collection and kairomone production*

For the production of fish kairomone, three-spined sticklebacks (*Gasterosteus aculeatus*) were collected from the ponds of the Ruhr-University Bochum. The Ruhr-University Bochum as a research facility has general permission that allows keeping of vertebrates for experimental purposes. For the production of the kairomone, fishes were not harmed and kept under conditions complying with care and welfare. After the experiment animals were released to the pond of origin.

A maximum of 20 fish no larger than 5 cm (body length) were kept in a 80 L glass aquarium at 15°C under constant 12∶12 h day∶night cycle. Animals were fed every 48 h with *Chironomus* larvae.

For the production of the kairomone one fish (size 4 to 5 cm body length) was transferred into 1 L water (Evian, Danone Waters, Germany) for 24 h. After the fish was removed, the water containing the kairomone was filtered (45 µm GF/C Whatman filter). In order to decrease bacterial degradation ampicillin (10 mg/l) (Sigma Aldrich, Germany) was added. Kairomone was frozen at −20°C and thawed before use. The concentration of one fish per liter was defined as 100% fish kairomone concentration.

In order to exclude an impact of ampicillin on the *Daphnia* development, we tested the effect of ampicillin in pre-trials. We did not observe any significant changes in life history, morphology or an increased survival rate.

### Induction assay

Age-synchronized mothers were randomly selected from the different cohorts. Only mothers holding black-eyed embryos i.e. 12 h before parturition were isolated in a glass dish. Embryos were dissected from the mother's brood pouch under a dissecting microscope (Olympus SZX 10). Randomly selected embryos were placed individually in 50 mL glass vials containing water (Evian; Danone, Germany) with a determined concentration of neurotransmitter and kairomone. To ensure constant stimulation, the culture medium was exchanged every 48 h.

Neurotransmitter and kairomone concentrations used were well below toxicity, which was ensured by determining the capability of the animals to molt into the next instar and eventually reach sexual maturity in pre-trials with concentration gradients (Fig. S1). Concentrations of GABA and picrotoxin did not increase the mortality. However, also high concentrations did not influence neckteeth expression. Concentrations were applied as published earlier [12].

The induction experiment contained different agonists and antagonists that were applied in combination with variable *Gasterosteus* (0%, 25%, 50%, 100%) or *Chaoborus* (0%, 25%, 50%, 100%) kairomone concentrations. Each experimental trial consisted of kairomone control treatments that did not contain neuro-effective drugs. In addition different GABA- and acetylcholine- receptor stimulants were combined with the different kairomone concentration. The neuro-effective drugs were prepared by serial dilution of the stock solution with ultrapure water (Genpure, Thermo Electron LED GmbH; Germany) or analytical grade ethanol (100%). The diluted stocks were mixed with Evian water (Danone Waters, Germany) to make a total volume of 40 ml. We stimulated the cholinergic system by using physostigmine (final concentration 5 nM, Sigma Aldrich, Germany, stock solutions (Stock A = 10 mM prepared in analytical grade ethanol (100%) and diluted (1∶100 in ultrapure water) to Stock B = 100 µM) were stored at −20°C) and inhibited with the antagonist atropine (final concentration 5 µM [12], Sigma Aldrich Germany, stock solution (500 µM) was prepared with ultrapure water and stored at −20°C). The gabaergic system was stimulated using GABA (final concentration of 15 µM, Sigma Aldrich, Germany, stock solution (10 mM) was prepared with ultrapure water and stored at −20°C, thawed prior to use) and inhibited using the antagonist picrotoxin (final concentration 0.1 µM and 0.5 µM [12]; Sigma Aldrich, Germany; stock solution (Stock A = 5 mM prepared in analytical grade ethanol (100%) and diluted (1∶10 in ultrapure water) to Stock B = 500 µM) was stored at −20°C). For every treatment we performed a minimum of 20 replicates.

We measured morphological defenses and the individual strength of neckteeth expression (as reported by Tollrian 1993 [9]) in the second juvenile instar. In order to determine life history shifts we measured body length at sexual maturity, counted the number of eggs and daily determined the time needed to obtain the first clutch. Body length measurements were performed using a dissecting microscope (Olympus SZX 12) in combination with a digital image analysis system (ColorView III and Cell∧D, Soft Imaging Solutions GmbH, Germany). Body size was determined from the upper eye margin to the junction of the carapace and the spine.

### Data analysis

Data of the predator effects (inducible defenses against *Chaoborus* and *Gasterosteus*) was acquired over a period of 18 months within timely synchronized experimental trials. No significant differences were detected (Kruskal-Wallis test) between the different trials and therefore individual data sets were pooled.

Statistical analyses were performed using Statistica 9.0 (Statsoft Europe, GmbH.). Neurotransmitter effects were determined by comparison of neurotransmitter treatments to the control.

The datasets were not normally distributed and thus analyzed using a non-parametric Mann-Whitney-U rank sum test to identify differences between groups. In case of multiple testing, p-values were Bonferroni-corrected.

## Supporting Information

Figure S1 **Toxicity Testing in Pre-trials.** A: Plotted is the mortality rate in dependence of physostigmine. EC 50 (the concentration at which 50% of the animals die) lies at 0.01 µM, which is double of the applied concentration that was used in the physiological induction assay. B: Plotted is the mortality rate in dependence of atropine. EC 50 lies above 10 µM and below 50 µM. The induction assay was therefore performed with a concentration of 5 µM atropine. C: Plotted is the mortality rate in dependence of picrotoxine. EC 50 could not be determined with the applied concentrations. However, picrotoxine did not impact neckteeth expression at all concentrations measured (E). D: Plotted is the mortality rate in dependence of the GABA. EC 50 could not be determined with the applied concentrations. However, GABA did not impact neckteeth expression at all concentrations measured (F). E: In the absence of *Chaoborus* (0%) cues, neckteeth expression is not influenced by picrotoxine (Kruskal-Wallis (4; 36) = 4; p = 0.9) *Chaoborus* (100%) induced neckteeth expression in dependence of the picrotoxine concentration. Neckteeth are not enhanced also at high concentrations (Kruskal-Wallis (4; 44) = 3.93; p = 0.41). F: In the absence of *Chaoborus* (0%) cues, neckteeth expression is not influenced by GABA (Kruskal-Wallis (4; 60) = 4; p = 0.4) *Chaoborus* (100%) induced neckteeth expression in dependence of the GABA concentration. Neckteeth are not inhibited also at high concentrations (Kruskal-Wallis (4; 29) = 7.96; p = 0.09).
(TIF)
