# Supplementary figures and images for: Chaoborus and Gasterosteus Anti-Predator Responses in Daphnia pulex Are Mediated by Independent Cholinergic and Gabaergic Neuronal Signals
Source: PLoS One. 2012 May 9;7(5):e36879. doi: 10.1371/journal.pone.0036879 (PMC3348892; doi:10.1371/journal.pone.0036879)

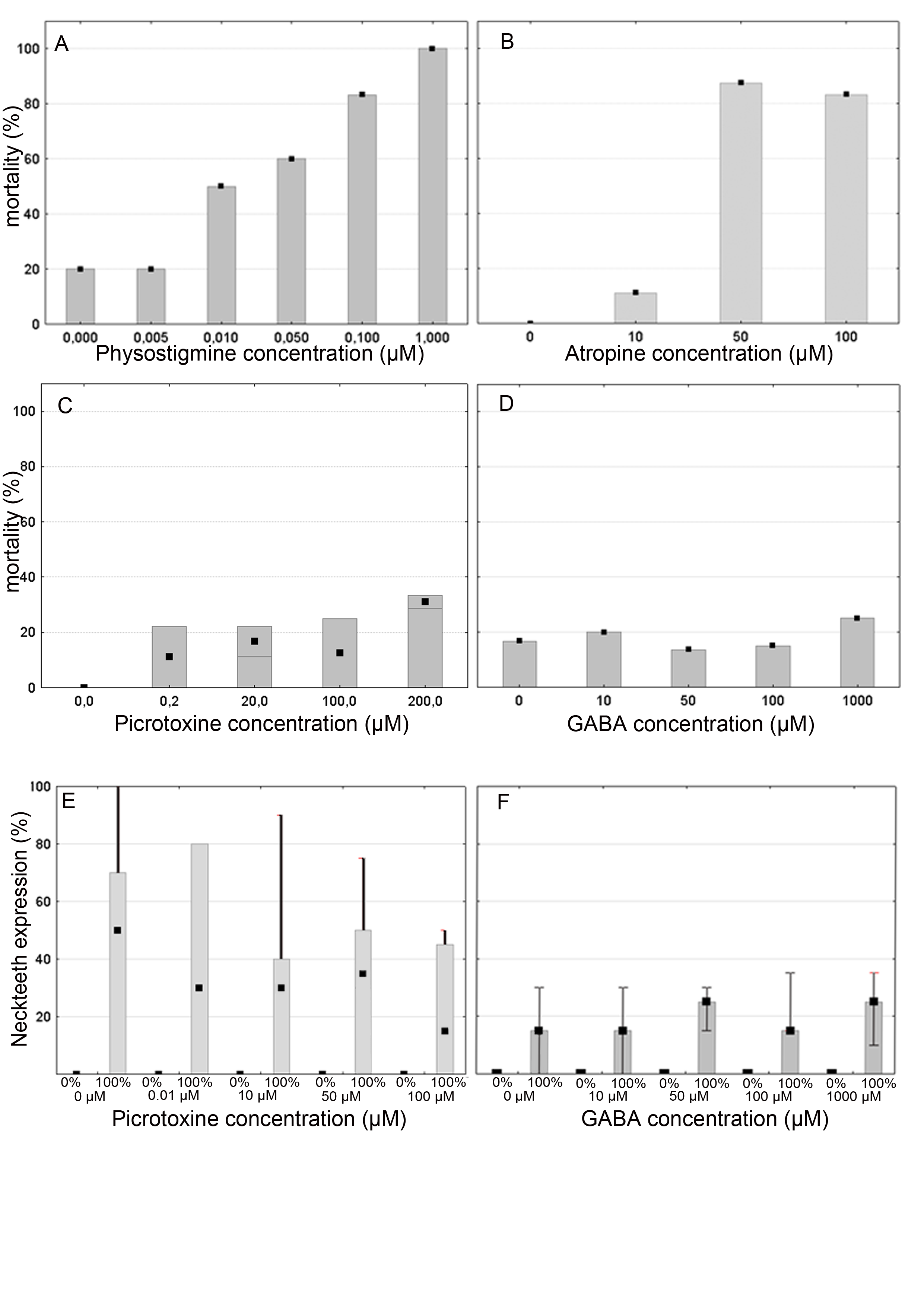

Supplement: Figure S1 — Toxicity Testing in Pre-trials. A: Plotted is the mortality rate in dependence of physostigmine. EC 50 (the concentration at which 50% of the animals die) lies at 0.01 µM, which is double of the applied concentration that was used in the physiological induction assay. B: Plotted is the mortality rate in dependence of atropine. EC 50 lies above 10 µM and below 50 µM. The induction assay was therefore performed with a concentration of 5 µM atropine. C: Plotted is the mortality rate in dependence of picrotoxine. EC 50 could not be determined with the applied concentrations. However, picrotoxine did not impact neckteeth expression at all concentrations measured (E). D: Plotted is the mortality rate in dependence of the GABA. EC 50 could not be determined with the applied concentrations. However, GABA did not impact neckteeth expression at all concentrations measured (F). E: In the absence of Chaoborus (0%) cues, neckteeth expression is not influenced by picrotoxine (Kruskal-Wallis (4; 36) = 4; p = 0.9) Chaoborus (100%) induced neckteeth expression in dependence of the picrotoxine concentration. Neckteeth are not enhanced also at high concentrations (Kruskal-Wallis (4; 44) = 3.93; p = 0.41). F: In the absence of Chaoborus (0%) cues, neckteeth expression is not influenced by GABA (Kruskal-Wallis (4; 60) = 4; p = 0.4) Chaoborus (100%) induced neckteeth expression in dependence of the GABA concentration. Neckteeth are not inhibited also at high concentrations (Kruskal-Wallis (4; 29) = 7.96; p = 0.09). (TIF) [file pone.0036879.s001.tif]
